# The Yunnan national medicine Maytenus compound inhibits the proliferation of hepatocellular carcinoma (HCC) by suppressing the activation of the EGFR-PI3K-AKT signaling pathway

**DOI:** 10.7150/jca.56426

**Published:** 2021-04-07

**Authors:** Wen-Tao Zhao, Liu-Xin Han, Lin Liu, Bao-Zhen Zeng, Yi Zhang, Liu-Fang Zhao, Hong-Yan Hu, Jia-Wei Xia, Yi-Ze Li, Xu-Dong Xiang, Xiao-Lin Lin, Di Lu, Gao-Feng Li

**Affiliations:** 1 Department of Gastrointestinal Oncology, The Third Affiliated Hospital of Kunming Medical University (Yunnan Cancer Hospital, Yunnan Cancer Center), Kunming 650118, China.; 2 The third people's hospital of Kunming (The Sixth Affiliated Hospital of Dali University), Kunming 650041, China.; 3 Department of Traditional medicine research laboratory, Puer Traditional Ethnomedicine Institute, Puer 665000, China.; 4 Department of Pathology, Guangdong Provincial People's Hospital, Guangdong Academy of Medical Sciences, Guangzhou 510080, China.; 5 Department of Gynecology, The Third Affiliated Hospital of Kunming Medical University (Yunnan Cancer Hospital, Yunnan Cancer Center), Kunming 650118, China.; 6 Department of Head and Neck Cancer, The Third Affiliated Hospital of Kunming Medical University (Yunnan Cancer Hospital, Yunnan Cancer Center), Kunming 650118, China.; 7 Department of Pathology, The Third Affiliated Hospital of Kunming Medical University (Yunnan Cancer Hospital, Yunnan Cancer Center), Kunming 650118, China.; 8 Department of Thoracic Surgery, The Third Affiliated Hospital of Kunming Medical University (Yunnan Cancer Hospital, Yunnan Cancer Center), Kunming 650118, China.; 9 Guangdong Provincial Key Laboratory of Cancer Immunotherapy Research and Guangzhou Key Laboratory of Tumor Immunology Research, Cancer Research Institute, School of Basic Medical Sciences, Southern Medical University, Guangzhou 510515, China.; 10 Technology Transfer Center, Kunming Medical University, Kunming 650500, China.

**Keywords:** Maytenus compound, Hepatocellular Carcinoma (HCC), EGFR-PI3K-AKT signaling pathway, Proliferation

## Abstract

**Objective:** To investigate the effects of Maytenus compound on the proliferation of hepatocellular carcinoma (HCC) cells *in vitro* and *in vivo* and to explore the underlying mechanism.

**Methods:** The half maximal inhibitory concentration (IC50) values of Maytenus compound in HepG2 and BEL-7402 cells were determined by the MTS assay. HepG2 and BEL-7402 cells were treated with different concentrations of Maytenus compound. MTS assay, colony formation assay and cell cycle analysis were performed to clarify the inhibitory effect of Maytenus compound on the proliferation of HepG2 and BEL-7402 cells *in vitro*. After subcutaneous injection of HepG2 cells, nude mice were randomly divided into a vehicle control group and a drug intervention group, which were intragastrically administered ddH_2_O or Maytenus compound, respectively. The inhibitory effect of Maytenus compound on the proliferation of HepG2 cells *in vivo* was analyzed using subcutaneous tumor growth curves, tumor weight, the tumor growth inhibition rate and the immunohistochemical detection of BrdU-labeled cells in S phase. The organ toxicity of Maytenus compound was initially evaluated by comparing the weight difference and organ index of the two groups of nude mice. The main proteins in the EGFR-PI3K-AKT signaling pathway were detected by Western blot after Maytenus compound intervention *in vivo* and *in vitro*.

**Results:** Maytenus compound showed favorable antiproliferation activity against HepG2 and BEL-7402 cells with IC50 values of 79.42±11.71 µg/mL and 78.48±8.87 µg/mL, respectively. MTS assay, colony formation assay and cell cycle analysis showed that Maytenus compound at different concentration gradients within the IC50 concentration range significantly suppressed the proliferation of HepG2 and BEL-7402 cells *in vitro* and inhibited cell cycle progression from G1 to S phase. Additionally, Maytenus compound, at an oral dose of 2.45 g/kg, dramatically inhibited, without obvious organ toxicity, the proliferation of subcutaneous tumors formed by HepG2 cells in nude mice. In addition, the tumor growth inhibition rate for Maytenus compound was 66.94%. Furthermore, Maytenus compound inhibited the proliferation of liver orthotopic transplantation tumors in nude mice. Western blot analysis showed that Maytenus compound significantly downregulated the expression of p-EGFR, p-PI3K, and p-AKT and upregulated the expression of p-FOXO3a, p27, and p21 *in vivo* and *in vitro*.

**Conclusion:** Maytenus compound significantly inhibited the proliferation of HCC cells *in vitro* and *in vivo*. The downregulation of the EGFR-PI3K-AKT signaling pathway and subsequent inhibition of cell cycle progression from G1 to S phase is one of the possible mechanisms. Maytenus compound has a high tumor growth inhibition rate and has no obvious organ toxicity, which may make it a potential anti-HCC drug, but the results from this study need to be confirmed by further clinical trials in HCC patients.

## Introduction

Primary hepatocellular carcinoma (HCC) has an insidious onset and a high degree of malignancy. It is difficult to diagnose at the early stage, and patient prognosis is poor. HCC is among the malignant tumors with the highest morbidity and mortality rates in the world 1. Surgery and liver transplantation are the major treatment methods for HCC. However, 70%-80% of patients are at an advanced stage at the time of diagnosis 2. Surgery and liver transplantation are only suitable for 15% of HCC patients 3. Currently, the average five-year survival rate for HCC patients is less than 10% 4. Drugs for the systemic treatment for late-stage HCC are limited, and HCC is extremely insensitive to traditional chemotherapy drugs; thus far, there is no standard drug treatment or regimen 5. Sorafenib is a clinically recognized and effective therapeutic drug that targets HCC, and immunotherapy drugs, which have recently become a research focus, have also shown efficacy in HCC treatment to a certain extent. However, HCC can rapidly acquire drug resistance to sorafenib 6. Furthermore, clinical trial showed that the objective response rate (ORR) of sorafenib in the patients with HCC was only 2% to 3% 7. Thus, the survival of patients with advanced HCC is only prolonged by 2.8 months 8. Immunotherapeutic drugs are expensive, and clinical studies have shown that compared with placebo, the programmed death receptor-1 (PD-1) inhibitor pembrolizumab only prolongs the survival of HCC patients by 3 months 9. Therefore, the current treatment status of HCC is not optimistic, and the lack of effective therapeutic drugs is a substantial clinical challenge. Therefore, it is important to find new and effective drugs for HCC.

Maytenus compound is an innovative novel drug selected and developed from folk traditional medicine used by Lahu people in Yunnan Province, China, by the Pu'er Nationality Research Institute of Traditional Medicine in the process of exploring traditional Chinese medicine, and this drug has been granted a Chinese invention patent (patent number: ZL200710066377.7). Maytenus compound has been utilized for more than 500 years, and it has been included in Chinese Lahu Nationality Medicine and Selection of Lahu's Folk Traditional Medicine. Maytenus compound tablets consist of six herbal medicines, including Maytenus hookeri Loes, Oldenlandia Diffusa Willd, Balanophora Harlandii Hook.f, and Glycyrrhiza uralensis Fisch, and are prepared using aqueous extraction (sugar-coated tablet). As early as 2005, Maytenus compound was approved by the Yunnan Food and Drug Administration as a hospital formulation for clinical application (approval No: (Z) 05J02424). Maytenus compound tablets can be used for the clinical treatment of uterine fibroids and mammary gland hyperplasia, with efficacy rates as high as 90% and 98%, respectively, and few side effects 10. In the records of empirical prescriptions by the Lahu people, there is evidence of using Maytenus compound for the treatment of HCC. There is also a small sample single-center clinical observation study of Maytenus compound for the treatment of HCC in China. The study indicated that alpha-fetoprotein (AFP), a specific tumor marker, was significantly decreased in HCC patients after Maytenus compound treatment. Maytenus compound has an important role in improving the quality of life of HCC patients, but the underlying mechanism has not been elucidated 11. In our study, compared to Maytenus compound used in the aforementioned clinical studies, the drug used in our study had the same sovereign drug (Maytenus) but had the different compatible drugs, and the proportion of each component was different. The drug used in our study was easy to manufacture and inexpensive and could become a new drug candidate with potential research and development (R&D) and application prospects for HCC treatment. This study clarified the significant inhibitory effect of Maytenus compound on the proliferation of HCC cells both *in vitro* and *in vivo* and explored the possible mechanism of action to provide a theoretical basis for the application of Maytenus compound for the treatment of HCC in clinical practice.

## Materials and Methods

### Preparation of Maytenus compound

Per gram Maytenus compound tablet [(Z05J02424), Puer Traditional Ethnomedicine Institute, China] included Maytenus 235 mg, Sargrassum 176.5 mg, Oldenlandia Diffusa Willd 176.5 mg, Balanophora Harlandii Hook.f., 176.5 mg, Tripterygium Hypoglaucum Hutch 176.5 mg, and Paris Polyphylla 59 mg. The tablets were grinded into powder and dissolved in Dimethyl Sulfoxide (DMSO; D2650, Sigma, USA) for further tests. And the control is 0 µg/mL of Mytenus, which medium add with Dymethyl sulfoxide 12.

### Cell lines and cell culture

The human HCC cell lines HepG2 and BEL-7402 were purchased from the Cell Bank of the Chinese Academy of Sciences (Shanghai, China). The cells were cultured with Dulbecco's modified Eagle's medium (DMEM) (high glucose) containing 10% fetal bovine serum (FBS) in a humidified incubator with 5% CO_2_ at 37 °C. The Maytenus compound tablets were grinded into powder and dissolved in Dimethyl Sulfoxide (DMSO) before it was used to treat HCC cells.

### Determination of IC50

The experimental protocol was performed as described previously 12. Briefly, the HCC cells were seeded into 96-well culture plates (3×10^3^/well); each group had six duplicate wells. After the cells were cultured for 24 h, they were treated with different doses (0, 2.34375, 4.6875, 9.375, 18.75, 37.5, 75, 150, 300, 600, 1200 and 2400 µg/mL) of Maytenus compound for 72 h. Then, 30 µL of MTS (G111A, Promega, USA) was added to each well, and the cells were further incubated for 2 h. Absorbance of each sample was measured using a full wavelength scanner (Varioskan Flash, Thermo Fisher, USA) at 490 nm. IC50 is the concentration that is required to inhibit 50% of cell growth. The Bliss method was used to calculate the IC50 from survival curves 13.

### MTS assay and colony formation assay

For the MTS assay, the indicated cells were seeded in 96-well plates at 1× 10^3^ per well in a final volume of 200 μl of complete medium with different concentrations of Maytenus compound. For the colony formation assay, the cells were counted and seeded (200 cells/per well) in 6-well plates with complete medium for 24 h. Then, cells were treated with different concentrations of Maytenus compound for 72 h. Finally, cells were incubated continuously without the drug effect for 14 days.

### Cell cycle analysis

For the cell cycle analysis, HCC cells were seeded in 6-well plates (2×10^5^ per well) and cultured in complete medium in an incubator for 8 h. Then, the cells were cultured in complete medium with different concentrations of Maytenus compound for 24 h. The remaining experimental protocol was performed as described previously 14.

### *In vivo* antitumor tests

Female BALB/c nude mice (4-5 weeks old) were purchased from the Charles River Laboratory Animal Technology Inc., Beijing. HepG2 cells (2×10^6^ cells) were subcutaneously injected into the right armpit of nude mice (n=12). The mice were randomly divided into a control group (ddH_2_O, n=6) and a treatment group (2.45 g/kg of Maytenus compound, n=6). The dose (2.45 g/kg) was determined based on the results of the toxicological experiment by Puer Traditional Ethnomedicine Institute (Puer, China). The drug dose (2.45 g/kg ) refers to the dose of Maytenus compound tablets. The mice in the control group and treatment group were given ddH_2_O and Maytenus compound, respectively, intragastrically once per day. During the 30-day study period, tumor volume and body weight were measured every two days using a slide caliper and electronic scale. Tumor volume (mm^3^) was calculated as follows: volume=(D×d^2^)/2, where D is the longest diameter and d is the shortest diameter. To label the cells in the DNA synthesis phase (S phase), BrdU (30 mg/kg) was intraperitoneally injected into the mice 3 h before the mice were sacrificed. Tumors, liver, spleen and kidney were collected and weighed after the mice were sacrificed by cervical dislocation. Viscera index was calculated as follows: Viscera index = t/T × 100%, where t is the organ weight and T is the animal weight. Subcutaneous tumors extracted from mice in the control group and the drug treatment group were pooled based on group. In addition, protein was extracted for Western blot analysis.

To construct a liver orthotopic transplantation tumor model, HepG2 cells (6×10^6^ cells) mixed with Matrigel (Cat. No. 365234, BD) were subcutaneously injected into the liver of nude mice (n=20). Then, the mice were randomly divided into a control group (ddH_2_O) and a treatment group (2.45 g/kg of Maytenus compound). One week after liver orthotopic transplantation, Maytenus compound treatment was started. The mice in the control group and treatment group were given ddH_2_O and Maytenus compound, respectively, intragastrically once per day for 30 days. The liver was collected and analyzed after the mice were sacrificed. All animal protocols were approved by the Committee on Ethics in Animal Experiments of Kunming Medical University.

### Histological analysis and immunohistochemistry (IHC)

Histological analysis and IHC were performed as described previously 14. The primary antibody information is as follows: BrdU (GE Healthcare, dilution 1:50).

### Flow cytometry

HepG2 cells were seeded in 6-well plates at 2×10^5^ per well and cultured in complete medium in an incubator for 24 h. Then, the cells were treated with various concentrations of Maytenus compound (0, 20, 40 and 80 μg/mL) for 24 h. After treatment, the treated cells were harvested and washed with cold PBS buffer. The cells were resuspended in binding buffer and stained with 5 μL of annexin V-FITC plus 5 μL of propidium iodide (Beyotime, Shanghai, China) for 0.5 h at 4 °C in the dark. The stained cells were washed with binding buffer three times to remove excess stain and then resuspended in 500 μL of binding buffer. The percentage of apoptotic cells was analyzed using flow cytometry (BD, FACSCalibur, USA) within 1 h. The experiment was repeated three times.

### Western blot analysis

Protein lysates were separated on 10% SDS-PAGE gels and electrophoretically transferred to PVDF membranes (Millipore, ISEQ00010). Then, the blots were probed with primary antibodies against EGFR (CST, #4405), p-EGFR (CST, #4407), PI3K (Abcam, ab189403), p-PI3K (Abcam, ab182651), AKT (Abcam, ab8932), p-AKT (Abcam, ab179463), FOXO3a (Abcam, ab109629), p-FOXO3a (Abcam, ab154786), p27 (Abcam, ab32034), p21 (Abcam, ab109502), and alpha tubulin (Abcam, ab52866) overnight at 4°C, followed by incubation with an HRP-labeled horse anti-mouse IgG (CST, #7076) or goat anti-rabbit IgG (CST, #7074) for 1 h at room temperature. Signals were detected using enhanced chemiluminescence (ECL). Alpha tubulin was used as the protein loading control.

### Statistical analysis

Statistical analysis was conducted using the SPSS 17.0 software package and GraphPad Prism 8.0 software. Two-tailed Student's t-test was used for comparisons of two independent groups. The chi-square test was used to analyze the association between the control group and Maytenus compound treatment group in the liver orthotopic transplantation tumor model. Values were deemed statistically significant at *P<0.05, **P<0.01 and ***P<0.001.

## Results

### Maytenus compound inhibits the proliferation of HCC cells *in vitro*

**Figure 1:**
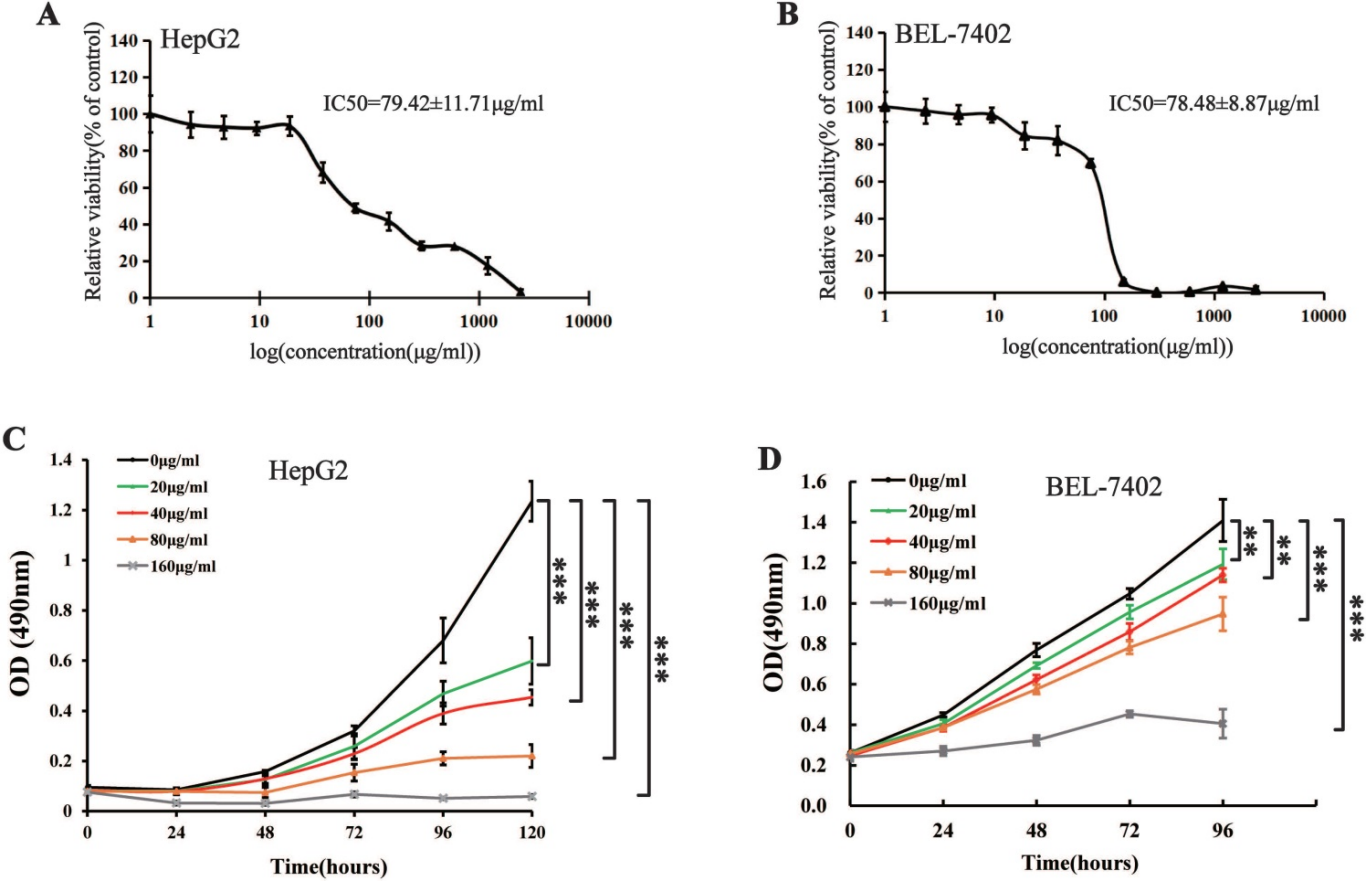
Effect of Maytenus compound on HepG2 and BEL-7402 cells *in vitro*. **(A-B)** IC50 of the Maytenus compound against HepG2 and BEL-7402 cells. **(C-D)** Cell proliferation of HepG2 and BEL-7402 cells treated with different concentrations of Maytenus compound was detected by MTS assay (**P < 0.01, ***P < 0.001).

**Figure 2:**
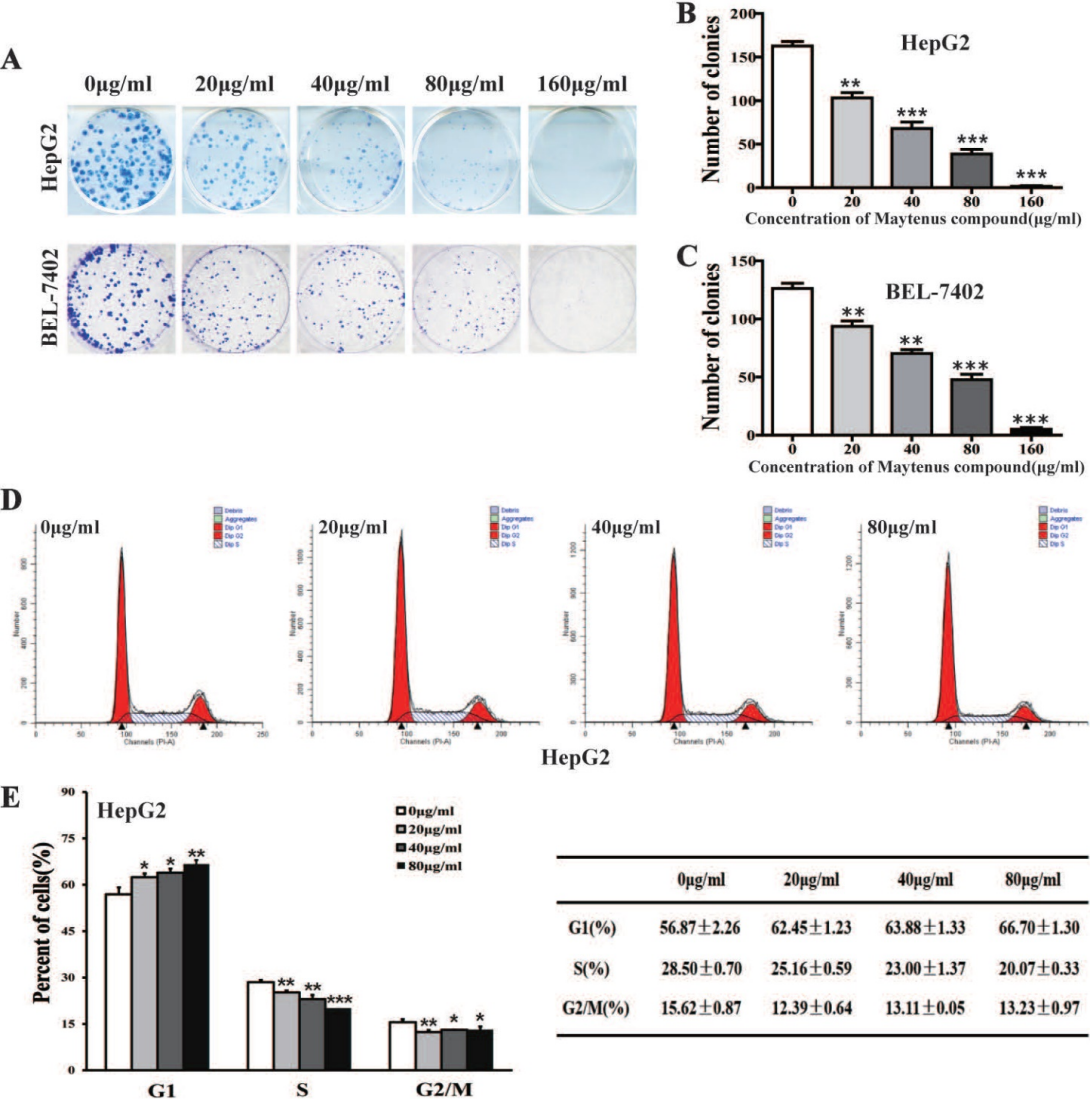
Maytenus compound inhibits the proliferation of HCC cells *in vitro*. **(A-C)** Maytenus compound inhibited clone formation in HepG2 and BEL-7402 cells with a dose-dependent mode. **(D-E)** Maytenus compound inhibited cell cycle progression from G1 phase to S phase in HepG2 cells with a dose-dependent mode (*P < 0.05, **P < 0.01, ***P < 0.001).

MTS cell proliferation, colony formation, and flow cytometry assays were performed to assess the inhibitory effect of different concentrations of Maytenus compound on the proliferation of HCC cells. The half maximal inhibitory concentration (IC50) values for Maytenus compound in HepG2 and BEL-7402 cells were 79.42±11.71 μg/mL and 78.48±8.87 μg/mL, respectively (Figure 1A-B). HepG2 and BEL-7402 cells were treated with Maytenus compound at different concentrations (0, 20, 40, 80 and 160 μg/mL) *in vitro* based on the IC50 values. The results of the MTS cell proliferation assay indicated that Maytenus compound significantly inhibited the proliferation of HepG2 and BEL-7402 cells in a dose-dependent manner (Figure 1C-D, *P*<0.001). Colony formation results indicated that different concentrations of Maytenus compound (0, 20, 40, 80, and 160 μg/mL) significantly inhibited the colony formation of HepG2 and BEL-7402 cells in a concentration-dependent manner (Figure 2A-C, *P*<0.01). The results of flow cytometry experiment showed that Maytenus compound, at each of the different concentrations tested (20, 40, and 80 μg/mL), significantly inhibited the cell cycle progression of HepG2 cells from growth 1 phase (G1 phase) to synthesis phase (S phase), leading to cell cycle arrest in G1 phase. In addition, the higher the concentration of Maytenus compound was, the more significant the G1 arrest (Figure 2D-E, *P*<0.05). The above results suggest that Maytenus compound can inhibit the proliferation of HCC cells *in vitro* through the inhibition of G1-S phase cell cycle progression.

### Maytenus compound inhibits the proliferation of HCC cells *in vivo*

**Figure 3:**
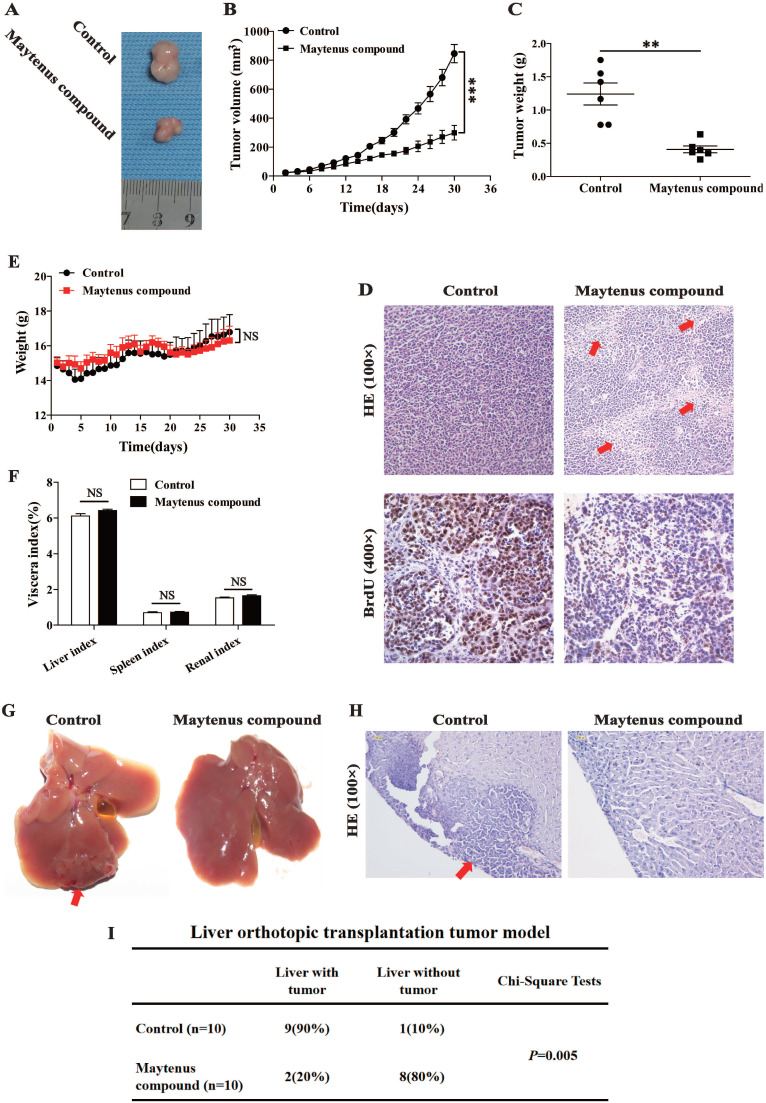
Maytenus compound inhibits HCC cell xenograft growth in nude mice. **(A)** Representative pictures of xenograft tumors treated with Maytenus compound and ddH2O, respectively (n=6/group). **(B)** Tumor growth curves of control group and Maytenus compound treatment group. **(C)** Tumor weight of control group and Maytenus compound treatment group. **(D)** Representative images of HE and BrdU staining of xenograft tumors of control group and Maytenus compound treatment group. The red arrow indicates cell necrosis. **(E)** The body weight of the nude mice in control group and Maytenus compound treatment group. **(F)** The viscera index of control group and Maytenus compound treatment group. **(G)** Representative pictures of liver orthotopic transplantation tumor treated with Maytenus compound and ddH_2_O, respectively. **(H)** Representative images of HE staining of liver orthotopic transplantation tumor of control group and Maytenus compound treatment group. The red arrow indicates the tumor formed in liver. **(I)** Liver tumor formation of the liver orthotopic transplantation tumor model after Maytenus compound treatment (**P < 0.01, ***P < 0.001).

Nude mice received a subcutaneous injection of HepG2 cells and were divided into two groups: mice in the control and drug intervention groups were given 2.45 g/kg/day of ddH_2_O and Maytenus compound, respectively, intragastrically for 30 days. The dose of Maytenus compound for intragastric administration was selected based on the results of toxicological experiments conducted in animals during the early stage of drug development. Maytenus compound significantly inhibited tumor proliferation in the mouse subcutaneous HCC tumor model (Figure 3). During the experiment, the volume and growth rate of subcutaneous tumors in the drug intervention group were significantly lower than those in the control group (Figure 3 A-B, *P*<0.001). Tumor weight in the drug intervention group was significantly lower than that in the control group (Figure 3 C, *P*<0.01). The calculated tumor inhibition rate for Maytenus compound was 66.94% [tumor inhibition rate=(tumor weight in control group-tumor weight in treatment group)/tumor weight in control group]. Hematoxylin and eosin (H&E) staining of subcutaneous carcinoma tissue sections showed that compared with the tumor tissue of the control group, a large amount of necrosis was found in the tumor tissue of the treatment group (Figure 3D). The number of BrdU-positive cells in the subcutaneous tumors in mice in the drug treatment group was significantly lower than that in mice in the control group (Figure 3D). The above results suggest that Maytenus compound suppresses the proliferation of HCC cells *in vivo* by decreasing the ratio of cells in S phase.

After establishing a liver orthotopic transplantation tumor model using HepG2 cells, mice were randomly divided into control and treatment groups. One week after liver orthotopic transplantation, ddH_2_O (2.45 g/kg/day) and Maytenus compound (2.45 g/kg/day) were intragastrically administered to mice in the control and treatment groups, respectively, once per day for 30 days. Tissues were collected and analyzed after 30 days of treatment. The results showed that among nude mice that received ddH_2_O, 90% exhibited tumor formation in the liver; in the treatment group, only 20% of nude mice exhibited tumor formation in the liver (Figure 3G-I). The difference between the two groups was significant (*P*=0.005). The results suggest that Maytenus compound can inhibit the *in situ* proliferation of HCC cells in the liver.

### Preliminary evaluation of the safety of Maytenus compound

In the course of intragastric administration, changes in the body weight of nude mice in the control and the treatment groups were measured and compared. The results showed that there was no significant difference in body weight between the two groups (Figure 3E). In addition, no statistically significant difference was observed in the vital organs (liver, spleen, and kidney) between the two groups (Figure 3F). These experimental results preliminarily demonstrate that Maytenus compound is safe and has no obvious organ toxicity.

### Maytenus compound promotes hepatoma cell apoptosis

**Figure 4:**
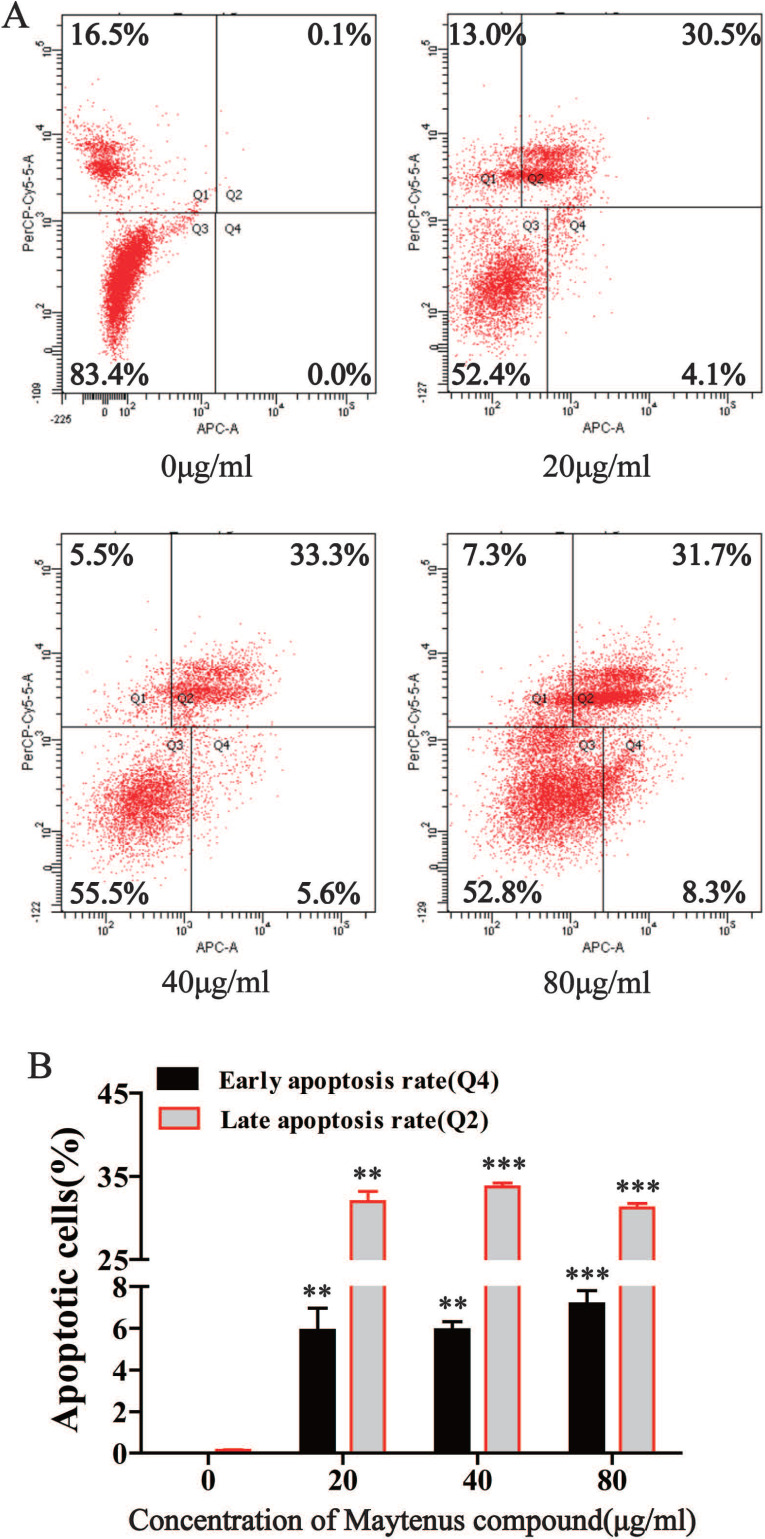
Maytenus compound promotes apoptosis of HCC cells *in vitro*. **(A)** Apoptotic cell ratio was detected by flow cytometry. **(B)** Maytenus compound increased the early and late apoptotsis rate of HepG2 cells with a dose-dependent mode (**P < 0.01, ***P < 0.001).

Flow cytometry results indicated that compared with the control group, different concentrations of Maytenus compound (20, 40, and 80 μg/mL) significantly increased the number of cells in early apoptosis (Q4) and late apoptosis (Q2) (Figure 4A and B, *P*<0.01). These results suggest that Maytenus compound can inhibit HCC cell proliferation by inducing cell apoptosis.

### Downregulation of the expression of key proteins in the EGFR-PI3K-AKT signaling pathway *in vitro* and *in vivo* by Maytenus compound

**Figure 5:**
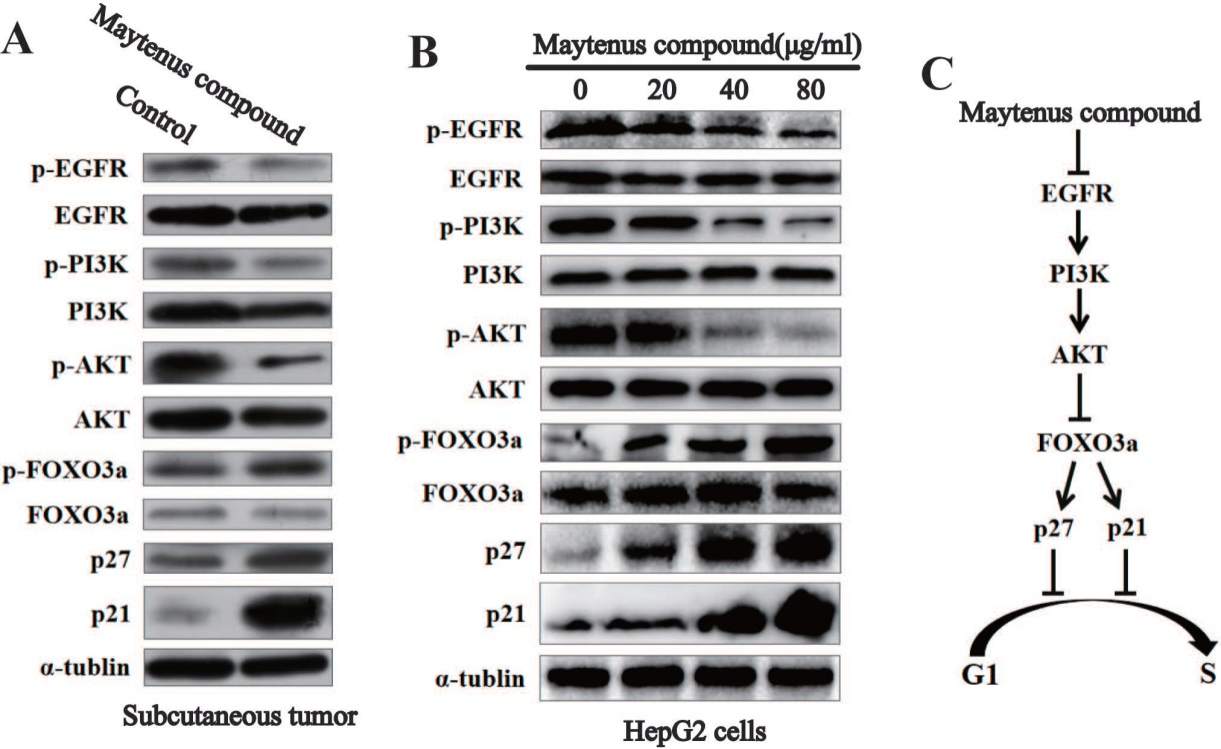
The EGFR-PI3K-AKT signaling pathway is down-regulated by Maytenus compound at the cell level *in vitro* and the animal level *in vivo*. **(A)** Maytenus compound suppresses EGFR-PI3K-AKT signaling pathway in subcutaneous tumor in nude mice. **(B)** Maytenus compound inhibits EGFR-PI3K-AKT signaling pathway in a dose-dependent manner in HepG2 cells. **(C)** A schematic diagram of the signal pathway that Maytenus compound inhibits HCC cell proliferation and induces cell cycle arrest via suppression of EGFR-PI3K-AKT pathway.

Western blot results for subcutaneous tissue from the control and treatment groups showed that Maytenus compound significantly downregulated the expression of phosphorylated EGF receptor (p-EGFR), phosphorylated phosphatidylinositol 3 kinase (p-PI3K), and phospho-AKT (p-AKT) in nude mice, thereby upregulating the protein expression of phosphorylated forkhead box class O 3a (p-FOXO3a), cyclin dependent kinase inhibitor p27 (p27), and p21 (Figure 5A). The Western blot results for HCC cells treated with different concentrations of Maytenus compound showed that Maytenus compound downregulated the expression of key proteins in the EGFR-PI3K-AKT signaling pathway *in vitro* and that the downregulation amplitude was positively correlated with drug concentration (Figure 5B).

## Discussion

HCC is highly malignant, and HCC patients have a poor prognosis. There are limited treatment methods and drugs for HCC. Therefore, there is an urgent need for effective drugs. Traditional Chinese medicine has been used for cancer treatment for thousands of years, with a great accumulation of knowledge by the people who uses such treatment. With the development of traditional Chinese medicine, some Chinese herbal preparations or extracts, such as artemisinin, have been confirmed to have significant anticancer activity without obvious adverse side effects 15, 16. In this study, the Yunnan national medicine Maytenus compound also showed significant anti-HCC activity without significant organ toxicity.

Previous studies have shown that antitumor substances isolated from Maytenus compound developed in Yunnan are mainly maytansine and maytanprine 17. Cell-based studies have shown that maytansine is nearly 100-fold more cytotoxic than vinca alkaloids and exhibits highly potent antitumor activity 18. Therefore, as early as the 1970s and 1980s, a great number of clinical studies attempted to apply maytansine to clinical treatments. Unfortunately, positive results for maytansine in the treatment of colorectal cancer 19 and melanoma 20, lymphoma 21, breast cancer 22, small cell lung cancer 23, soft tissue sarcoma 24, cervical cancer 25, and pancreatic cancer 26 were not obtained in phase II clinical studies primarily because maytansine has poor water solubility and high toxicity and is nonselective 18, 27, which led to extremely low dose-limiting toxicities (2 mg/m^2^) in the human body. Therefore, the manifestation of antitumor activity was not possible using a tolerable dose in humans 28. The use of Maytenus as a traditional Chinese medicine preparation can reduce the dose-limiting toxicity of Maytenus folk prescriptions without reducing the efficacy of Maytenus compound preparations. This observation provides a new research idea for advancing the application of Maytenus compound, which contains maytansine and maytanprine, to clinical practice. Studies have shown that the efficacy of traditional Chinese medicine compound preparation results from the joint action of various ingredients of each medicine 29. Maytenus compound is a Chinese herbal compound composed of six substances in accordance with the principle of monarch, minister, assistant and guide, and the inhibition of HCC proliferation results from the joint action of various ingredients.

The results at the cell level showed that the different drug concentrations of Maytenus compound significantly inhibited the proliferation of HCC cells *in vitro* and inhibited cell cycle progression from G1 to S phase in HCC cells. In animal studies, BrdU was used to label cells at S phase. Because BrdU is an analog of thymidine, BrdU can be incorporated into newly synthesized DNA when cells are in the DNA synthesis phase (S phase). Therefore, BrdU-positive cells are cells in S phase. Immunohistochemistry results showed that the number of BrdU-positive cells (cells in the S phase) in the subcutaneous tumors in mice in the treatment group was significantly less than that in positive control cells from mice in the control group, indicating that Maytenus compound significantly inhibited cell cycle progression from G1 to S phase in HCC cells *in vivo.* The results from the cell experiments and animal studies are mutually reinforcing. To closely simulate the actual anti-HCC activity of Maytenus compound *in vivo*, we used a liver orthotopic transplantation tumor model in nude mice. Using this model, we confirmed that this drug inhibited the *in situ* proliferation of HCC cells *in vivo*. These results fully confirmed that Maytenus compound exhibits significant antiproliferative effects on HCC both *in vitro* and *in vivo*. More importantly, the drug used in this study is easy to produce and inexpensive and has been used for many years in clinical practice to treat mammary gland hyperplasia and uterine fibroids, confirming the good safety profile of this drug. These results strongly suggest that Maytenus compound can be a potential drug for the clinical treatment of HCC.

Epidermal growth factor receptor (EGFR) is a transmembrane tyrosine kinase receptor that can be activated by a variety of ligands and subsequently activate multiple signaling pathways that control proliferation, differentiation and survival 30, thereby promoting tumor cell proliferation, metastasis, and the inhibition of cell apoptosis 31. Many studies have shown that the EGFR-PI3K-AKT signaling axis plays an important role in the malignant progression of HCC 31, 32. Consistent with the above findings, Western blotting was used to detect changes in the protein levels of p-EGFR, EGFR, p-PI3K, PI3K p-AKT, AKT, p-FOXO3a, FOXO3a, p27 and p21 in subcutaneous tumor tissue and HepG2 cells treated with Maytenus compound. The results showed that Maytenus compound downregulated the EGFR-PI3K-AKT signaling pathway at the cell culture level and in the animal model, further inhibiting cell cycle progression from G1 to S phase and thereby inhibiting HCC cell proliferation. Flow cytometry results showed that Maytenus compound induced HCC cell apoptosis. These results indicated that the anti-HCC effect of Maytenus compound was the result of the combined effects of the inhibition of proliferation and the promotion of apoptosis. The findings in this study that the expression of the EGFR-PI3K-AKT signaling pathway can be downregulated by Maytenus compound is also consistent with those of a previous study of Maytenus compound in lung cancer and cervical cancer cells 12.

We found that the Maytenus compound may inhibit HCC cell proliferation by downregulating the EGFR-PI3K-AKT signaling pathway, but its specific direct targets are unknown, requiring further studies. Studies have shown that many traditional Chinese medicines generate anticancer activities through multiple targets 33, 34. Therefore, there are other possible molecular mechanisms by which Maytenus compound inhibits HCC proliferation. In summary, this study confirms that Maytenus compound exhibits significant anti-HCC effects both *in vitro* and *in vivo* and revealed a possible mechanism of action, providing a theoretical basis for the clinical use of Maytenus compound as a therapeutic drug for HCC. However, the application of Maytenus compound in HCC patients must be further investigated through clinical trials.
